# Study on the COVID-19 infection status, prevention and control strategies among people entering Shenzhen

**DOI:** 10.1186/s12889-021-10548-8

**Published:** 2021-03-20

**Authors:** Xuan Zou, Zi-Qian Xu, Bi-Xin Wang, Jian-Fan He, Jing-Zhong Wang

**Affiliations:** 1 Shenzhen Center for Disease Control and Prevention, Shenzhen, China; 2 Shenzhen Bay Laboratory, Shenzhen, China

**Keywords:** COVID-19, SARS-CoV-2, Imported cases, Prevention and control, Closed-loop management

## Abstract

**Background:**

The novel coronavirus disease 2019 **(**COVID-19) confirmed cases overseas have continued to rise in the last months, and many people overseas have chosen to return to China. This increases the risk of a large number of imported cases which may cause a relapse of the COVID-19 outbreak. In order to prevent imported infection, the Shenzhen government has implemented a closed-loop management strategy using nucleic acid testing (NAT) for severe acute respiratory syndrome coronavirus 2 (SARS-CoV-2) and requiring 14 days of medical observation for individuals with an overseas tour history (Hong Kong, Macao, Taiwan province and other countries). Our study aims to describe the status of COVID-19 infection among people entering Shenzhen, and to evaluate the effect of the closed-loop management strategy.

**Methods:**

We undertook a descriptive study and risk analysis by the entry time, time of reporting, and local confirmed cases in countries of origin. The NAT were completed in Shenzhen Center for Disease Control and Prevention (CDC), ten district-level CDCs, and fever clinics.

**Results:**

A total of 86,844 people from overseas entered Shenzhen from January 1 to April 18, 2020; there were 39 imported COVID cases and 293 close contacts. The infection rate of people entering was 4.49‰ [95% Confidence interval (CI): 3.26‰–6.05‰]. Fourteen imported cases (35.9%) came from the UK, and nine (23.08%) came from the USA. People entering from the USA since March 9 or from the UK since March 13 are the high-risk population. As of July 17, there have been no new confirmed cases in Shenzhen for 153 days, and the numbers of confirmed case, close contacts, and asymptomatic cases are 0.

**Conclusions:**

The closed-loop management has been effective in preventing imported infection and controlling domestic relapse. The distribution of entry time and report time for imported cases overseas was similar. This shows that it is important to implement closed-loop management at the port of entry.

## Background

In December 2019, the novel coronavirus disease 2019 (COVID-19) caused by severe acute respiratory syndrome coronavirus 2 (SARS-CoV-2) emerged from Wuhan, China. China reported the disease to the World Health Organization (WHO) and issued reports to other countries immediately [1, 2]. COVID-19 is characterized by fever, cough, fatigue, shortness of breath, pneumonia, and other respiratory tract symptoms [3, 4]. Infection via respiratory droplets or secretions from infected individuals is thought to be the predominant mode of human-to-human transmission [5–8]. The absence of fever with SARS-CoV-2 is more frequent than in SARS-CoV (1%) and Middle East respiratory syndrome coronavirus (MERS-CoV) infections (2%), so afebrile patients may be missed, and it is common to have asymptomatic cases [9]. No specific antiviral therapies are available, and efforts to develop antiviral drugs and a vaccine continue [10]. All these factors have caused difficulties in the prevention and control of COVID-19. The policies of “early detection, early report, early diagnosis, early quarantine and early treatment”, and “concentrate patients, concentrate experts, concentrate resources, concentrate treatment” have been effective in preventing and controlling the spread of COVID-19 in China, with the cooperation of government and society departments. After 76 days, China lifted the lockdown on Wuhan and allowed people to leave the city on April 8, 2020. The number of severe cases has been 0 from April 24, and the number of hospitalized cases has been 0 from April 26. The nationwide intervention to control COVID-19 had a real effect.

However, the overseas confirmed cases continued to rise. On March 11, 2020, WHO declared that the outbreak of COVID-19 could be characterized as a pandemic [11]. As of April 18, 2020, there were more than 2.1 million confirmed cases of COVID-19, and more than 140,000 deaths reported worldwide [12]. The first imported case in China was reported in Shenzhen on March 1, 2020. On April 25, the number of outbreaks in clusters associated with imported cases was about 60, which affected more than 10 provinces, including Guangdong. Shenzhen is situated in Guangdong province and at the border with Hong Kong, and there are many person-to-person exchanges, overseas students and transnational workers. As economic activity continues to resume in the coming weeks, the main aim of control has turned to preventing cases being imported from overseas and domestic relapse. In order to prevent imported infection, the Shenzhen government has implemented a closed-loop management strategy, with the cooperation of customs, immigration, the health bureau and other departments, by using nucleic acid testing (NAT) for SARS-CoV-2 and requiring 14 days of medical observation for individuals with an overseas tour history (Hong Kong, Macao, Taiwan province and other countries). Our study aimed to describe the status of COVID-19 infection among people entering Shenzhen, and evaluated the effect of the closed-loop management strategy.

## Methods

### Closed-loop management

On March 1, 2020, the Shenzhen Center for Disease Control and Prevention (CDC) reported the first imported case of COVID-19 from the United Kingdom. In order to prevent imported infection, the Shenzhen government has implemented a closed-loop management strategy, with the cooperation of customs, immigration, health bureau and other departments, using nucleic acid testing (NAT) for SARS-CoV-2 and requiring 14 days of medical observation for individuals with an overseas tour history (Hong Kong, Macao, Taiwan province and other countries).

**Fig. 1:**
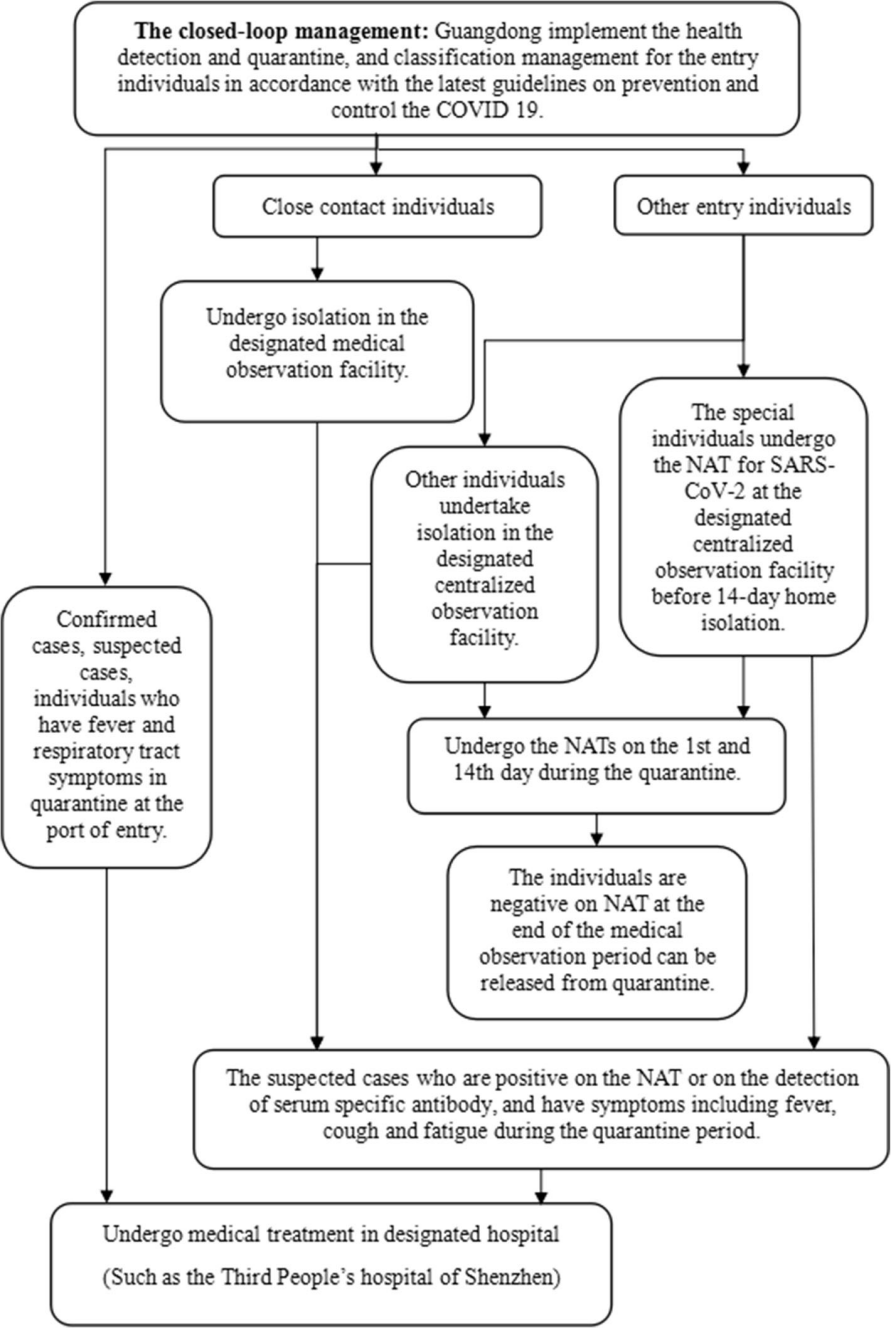
Flowchart of the closed-loop management system in Shenzhen

The first step is to divide the people entering from overseas into three categories for management, according to the Prevention and Control Plan for Coronavirus Disease 2019 (the sixth Edition) [13] (Fig. 1): 1. Confirmed cases, suspected cases, and individuals who have fever and respiratory tract symptoms in quarantine at the port undergo medical treatment in a designated hospital (such as the Third People’s Hospital of Shenzhen). 2. Close contact individuals undergo isolation in a designated centralized medical observation facility. They receive medical treatment in the designated hospital if they are positive on the NAT or on the detection of serum specific antibody, and have symptoms including fever, cough and fatigue during the quarantine period. 3. The special individuals who are under 14 or over 70 years old, pregnant women, or those not suitable for centralized observation with basal disease undertake home isolation for 14 days, but they are required to undergo the NAT for SARS-CoV-2 at the designated centralized medical observation facility before home isolation. The trinity cooperation community work group manages the health surveillance of people in home quarantine. The trinity cooperation community work group includes medical staff from community health service centers, community workers, and community police. They can provide maintenance of order, medical and life-saving services. They also have been screening the people entering Shenzhen from January 1, 2020 in every neighborhood. Other individuals entering Shenzhen undergo isolation in the centralized observation facilities.

Individuals can be released from quarantine if they are negative on the NAT at the end of the medical observation period. The suspected cases who are positive on the NAT or in the detection of serum specific antibody, and have symptoms such as fever, cough and fatigue during the quarantine period receive medical treatment in the designated hospital. Close contacts are identified through contact tracing of a confirmed, suspected or asymptomatic case and are defined as those who did not take effective protective measures and had contact with an index case 2 days before symptom onset, according to the Prevention and Control Plan for Coronavirus Disease 2019 (the sixth Edition). This kind of closed-loop management can ensure that entering people have no chance to make contact with residents, to prevent imported COVID-19 infection within the 14-day medical observation period.

The Guangdong–Hong Kong–Macao Greater Bay Area (GBA) has implemented mutual recognition measures for their medical quarantine observation results. Individuals who are negative on NAT at the end of medical observation can receive notification and be released from quarantine. The notification issued by local primary medical and health institutions has mutual recognition in GBA. The trinity cooperation community work group of the neighborhood committee manages the individuals who have completed their medical observation period in Hong Kong or Macao by providing health and consultation services, undertaking temperature surveillance, checking the self-health declaration, and sending those who have the symptoms of fever, cough, and fatigue to the designated hospital.

### Data sources and data analysis

This was a descriptive study, supported by the Shenzhen government and based on their policies. The government shared the data (from January 1 to April 18, 2020.) with us, so our data are secondary and public data, and can be referenced [14]. The individuals who have a history of travel (Hong Kong, Macao, Taiwan province and other countries) within 14 days undergo NAT for SARS-CoV-2 by reverse transcription–polymerase chain reaction (RT-PCR) of nasal swabs at 52 fever clinics, ten district-level CDCs, and Shenzhen CDC.

Based on the assumption that the emigrating population conforms to the distribution of the population of origin, we believe that the incidence in the population of origin can represent the risk in the emigrating population. Thus, we can calculate the importation risk (Risk _in_).

$$ {Risk}_{in}=\frac{Case}{Pop_1}\times \frac{Pop_2}{Pop_3}\times c $$

Case means the number of new confirmed cases in the origin per day, and the case data were derived from WHO Novel coronavirus (2019-NCoV) situation reports [15]. Pop_1_ means the size of the population in the location of origin, and the data came from the origin country’s official website [16–18]. Pop_2_ means the number of people entering Shenzhen from the origin per day. Pop_3_ means the total number of people entering Shenzhen per day. Pop_2_ and Pop_3_ were obtained from the Shenzhen government [19]. C is the constant and is valued at 1,000,000.

## Results

**Fig. 2:**
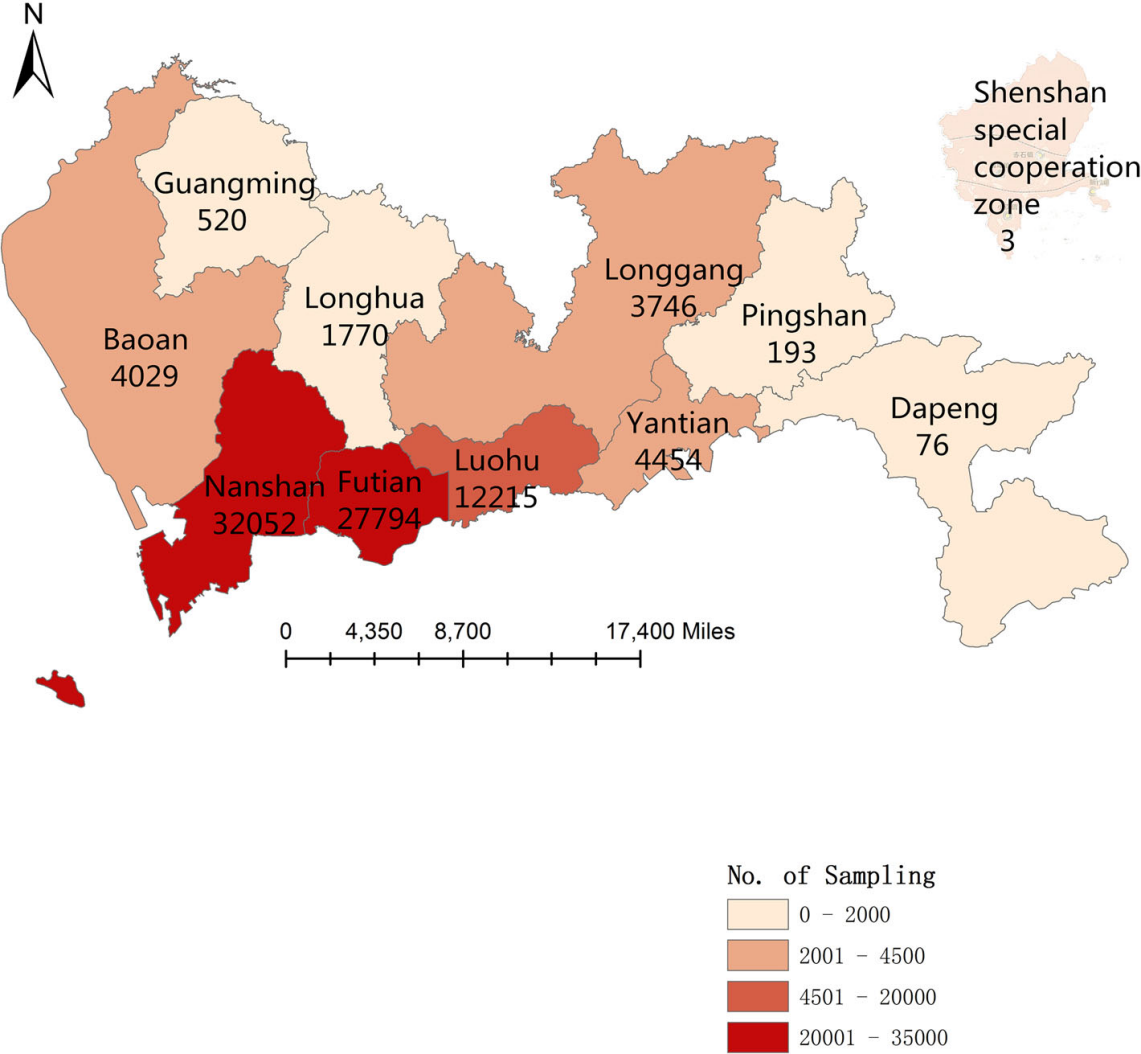
The distribution of all people entering Shenzhen

A total of 86,844 people from overseas entered Shenzhen from January 1 to April 18, 2020, and 77.09% have Chinese nationality. The remaining 22.91% have foreign nationalities. The mean age of the population was 32 years, and 52.6% were male. Figure 2 shows the distribution of these people in Shenzhen as produced by ARCGIS10.2, including the Shenshan special cooperation zone. The risk of imported infection in Nanshan, Futian and Luohu districts was higher than in others. The risk of imported infection in the southwest was higher than in northeast Shenzhen.

**Fig. 3:**
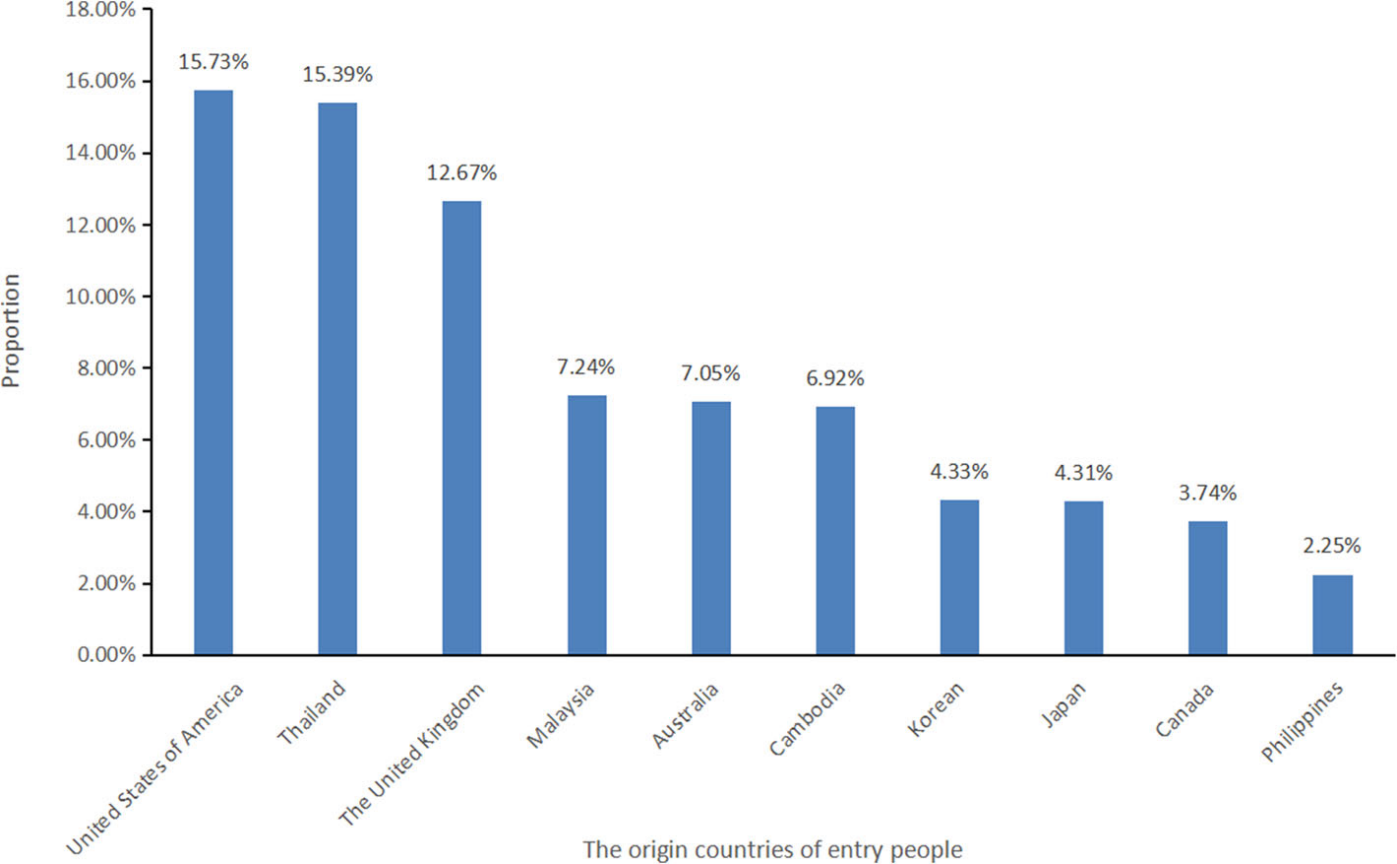
The top 10 countries of origin based on the number of people entering

**Fig. 4:**
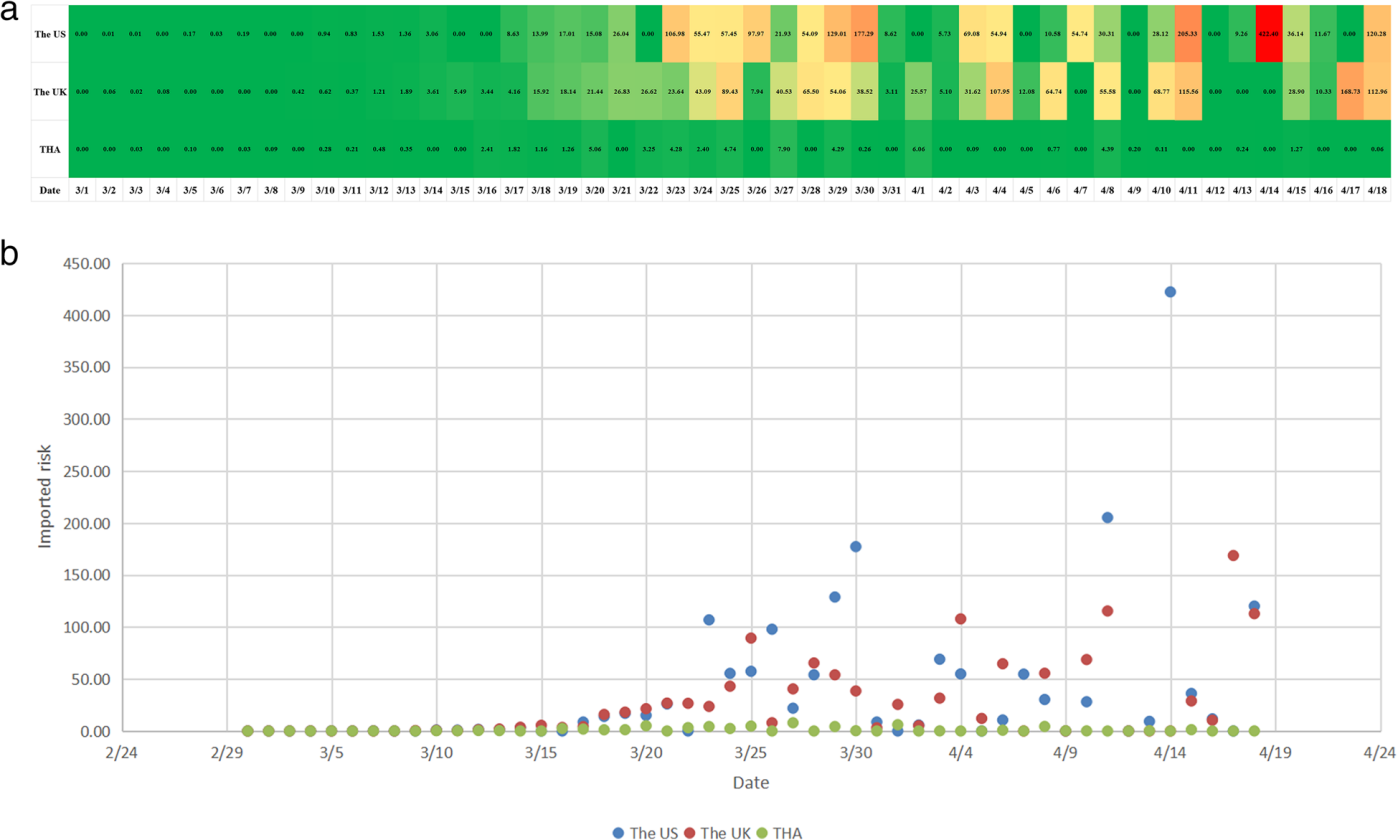
**a** Importation risk map (from January 3 to April 18). **b** Importation risk (From January 3 to April 18)

**Fig. 5:**
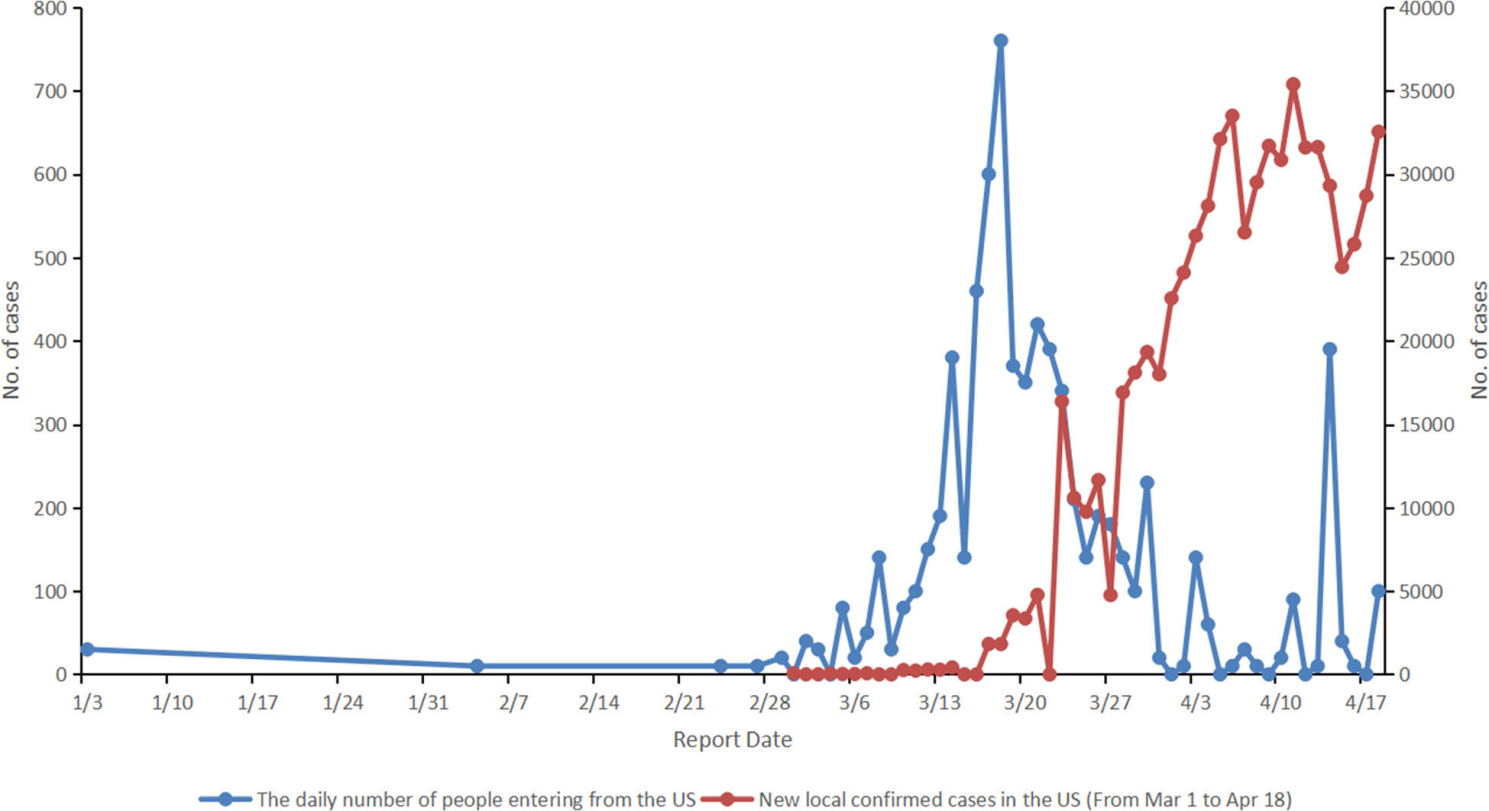
The daily number of people entering from the US (from January 3 to April 18) and new local confirmed cases in the US (from March 1 to April 18)

**Fig. 6:**
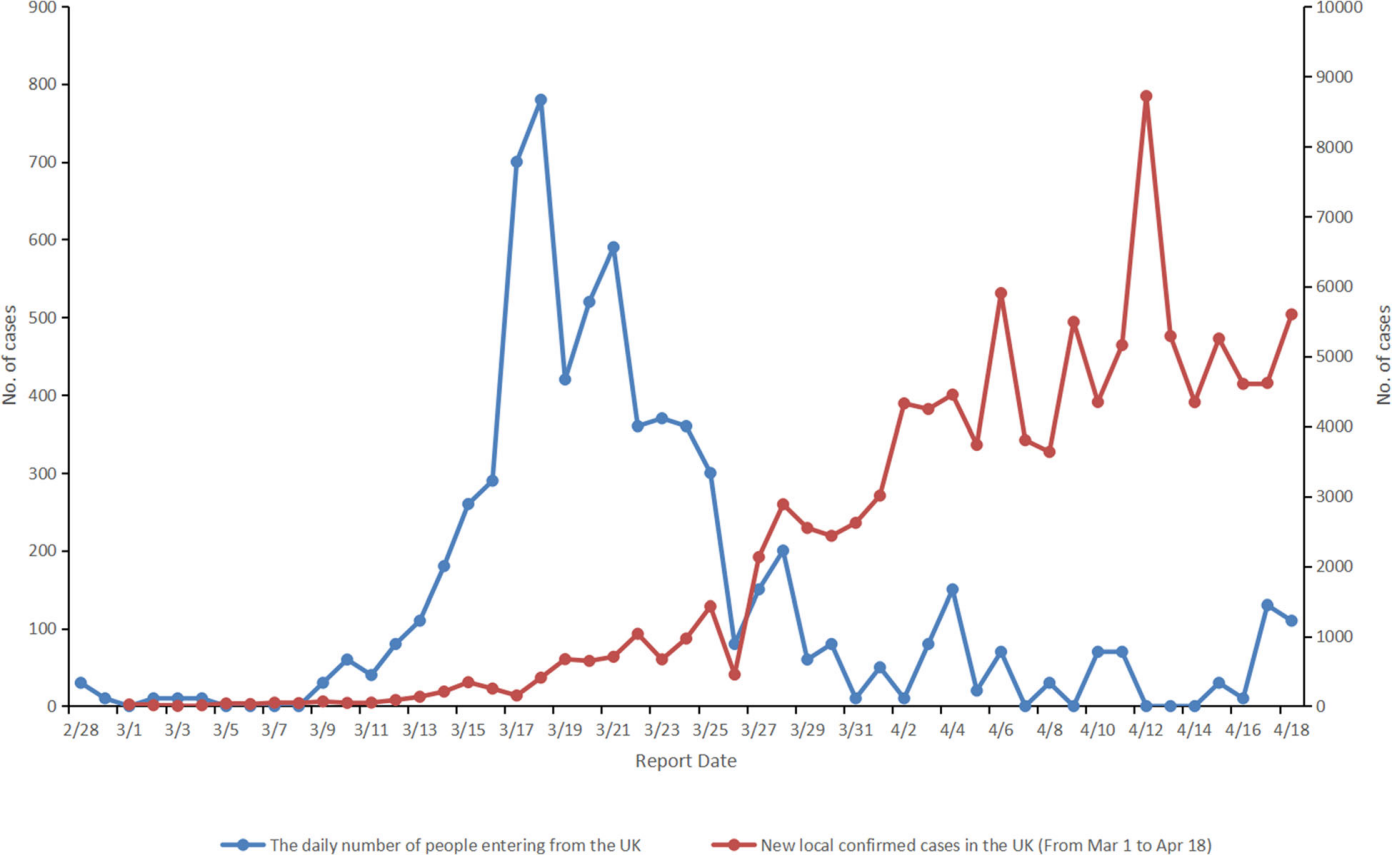
The daily number of people entering from the UK (from February 28 to April 18) and new local confirmed cases in the UK (from March 1 to April 18)

We ranked the countries of origin according to the number of people entering (Fig. 3): 15.73% of the population came from the United States of America (US), 15.39% came from Thailand (THA), and 12.67% came from the United Kingdom (UK). Subsequently, we analyzed the importation risk (Risk _in_) for the US, THA and the UK (Fig. 4) because the number of people entering from these top three countries was greater than from other places, so the risk of importation from these three countries is higher. Fig. 4 (a) and (b) show that the risk of importation from the US and the UK are higher than from THA. We therefore analyzed people from the US and UK and the local confirmed cases to produce Figs. 5 and 6. We excluded the data before March 1 because the number of people entering and new local confirmed cases were small in the countries of origin, and the importation risks were low.

Figure 5 indicates that there were two main peaks among people entering from the US, one peak concentrated on March 18, and another peak concentrated on April 14. The first confirmed case in the US was reported on January23, according to WHO Novel coronavirus (2019-NCoV) situation report 3 (accessed January 23, 2020). There were three main peaks among the new local confirmed cases in the US, the first concentrated on March 23 (16,354 confirmed new cases), the second concentrated on April 6 (33,510 confirmed new cases), and third concentrated on April 11 (35,386 confirmed new cases). We ignored the peak on April 18 because of incomplete data since April 18. More people entered from the US around March 18, and the new local confirmed cases reached the first main peak 5 days later, on March 23. The incubation period of COVID-19 can range from 1 to 14 days according to the WHO. The interval between March 9 and March 23 was 14 days, and there were still two peaks concentrated on April 6 and April 11. The people entering from the US since March 9 were a high-risk population for imported infection.

Figure 6 indicates that the entry of people from the UK was mainly concentrated in the period between March 13 and March 28, especially on March 18 and March 21, and there was a slight fluctuation after March 29. The first confirmed case in the UK was reported on February 2, according to WHO Novel coronavirus (2019-NCoV) situation report 13 (accessed February 2, 2020), and the first peak was on March 28 (2885 confirmed new cases). The interval between March 13 and 28 was 15 days, and there were several peaks over 8000 (April 12) since March 28. The people entering from the UK since March 13 were a high-risk population for imported infection.

As of April 18, 2020, there were 39 imported cases and 293 close contacts detected and reported; 82.05% of the imported cases were of Chinese nationality. The remaining 17.95% have foreign nationalities. The mean age of the population was 33 years, and 71.79% were male. The infection rate of individuals entering Shenzhen from January 1 to April 18, 2020 was 4.49‰ (95% confidence interval [CI], 3.26‰ to 6.05‰).

**Fig. 7:**
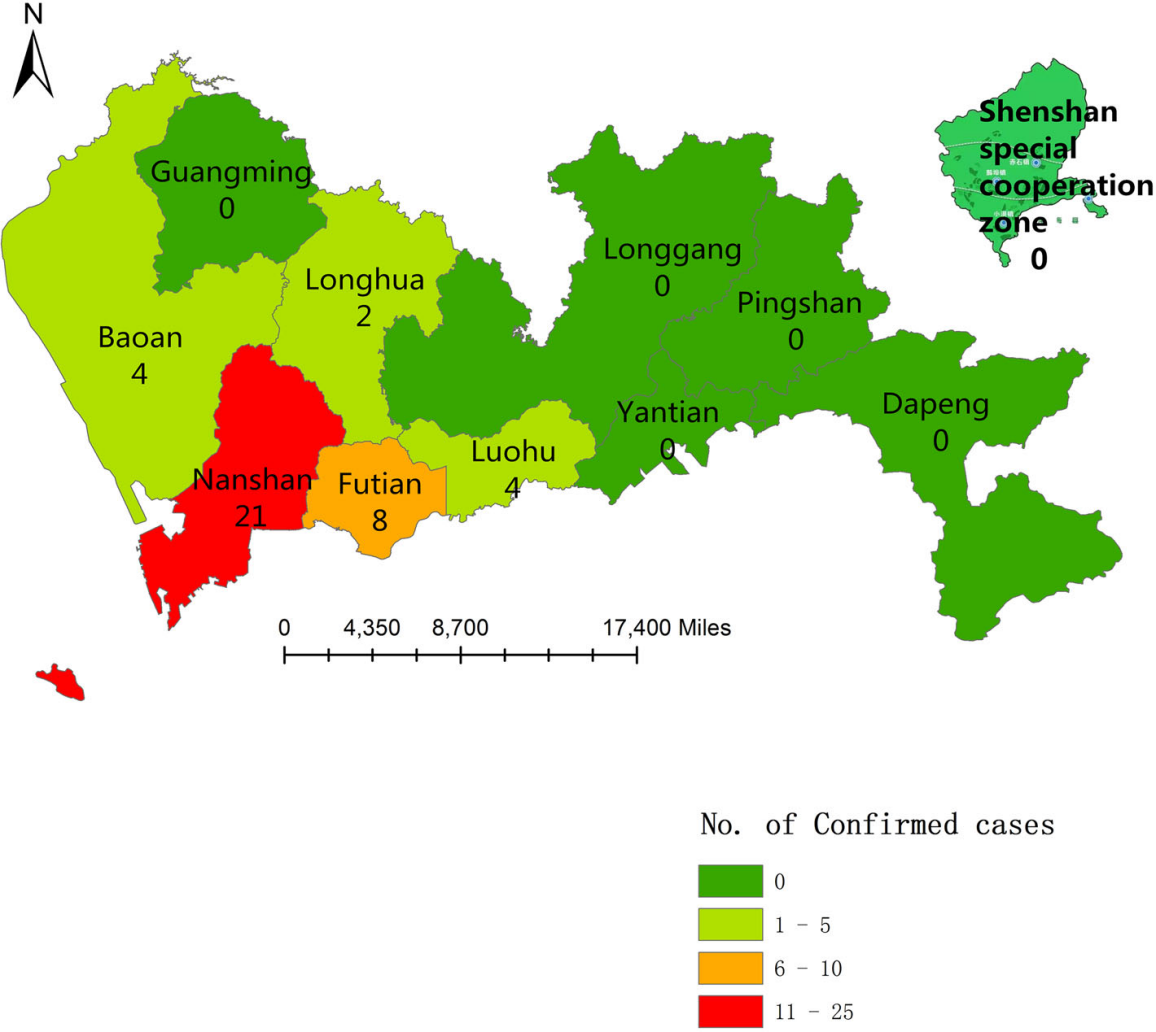
The distribution of imported cases in Shenzhen

Figure 7 shows the distribution of 39 imported cases in Shenzhen, produced by ARCGIS10.2, including the Shenshan special cooperation zone. The number of imported cases in Nanshan district was the highest in Shenzhen. All 39 imported cases and 293 close contacts underwent the closed-loop management with centralized medical observation when they entered Shenzhen; they received medical treatment in the designated hospital when they were positive on NAT. As of July 17, there have been no new confirmed cases in Shenzhen for 153 days, and the number of confirmed cases, close contacts, and asymptomatic cases is 0. This means the closed-loop management system has been effective in preventing imported infection and controlling domestic relapse.

**Fig. 8:**
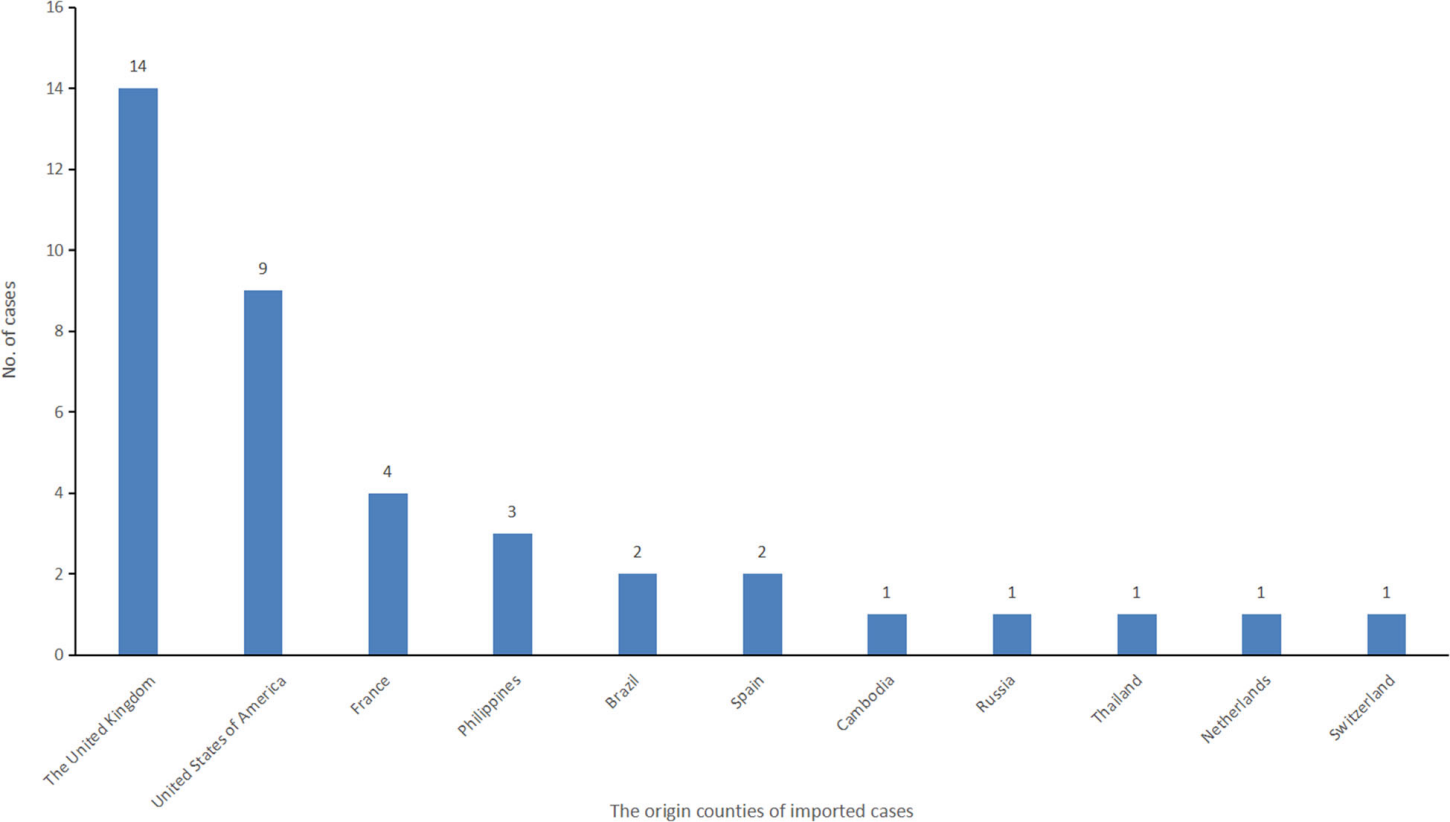
The countries of origin of imported cases

We ranked the countries of origin based on the number of imported cases (Fig. 8). There were 14 imported cases (35.9%) from the UK, 9 (23.08%) from the US, 4 (10.26%) from France, 3 (7.69%) from the Philippines, 2 (5.13%) from Brazil, and 2 (5.13%) from Spain. The imported cases in Shenzhen mainly came from the UK and the US.

**Fig. 9:**
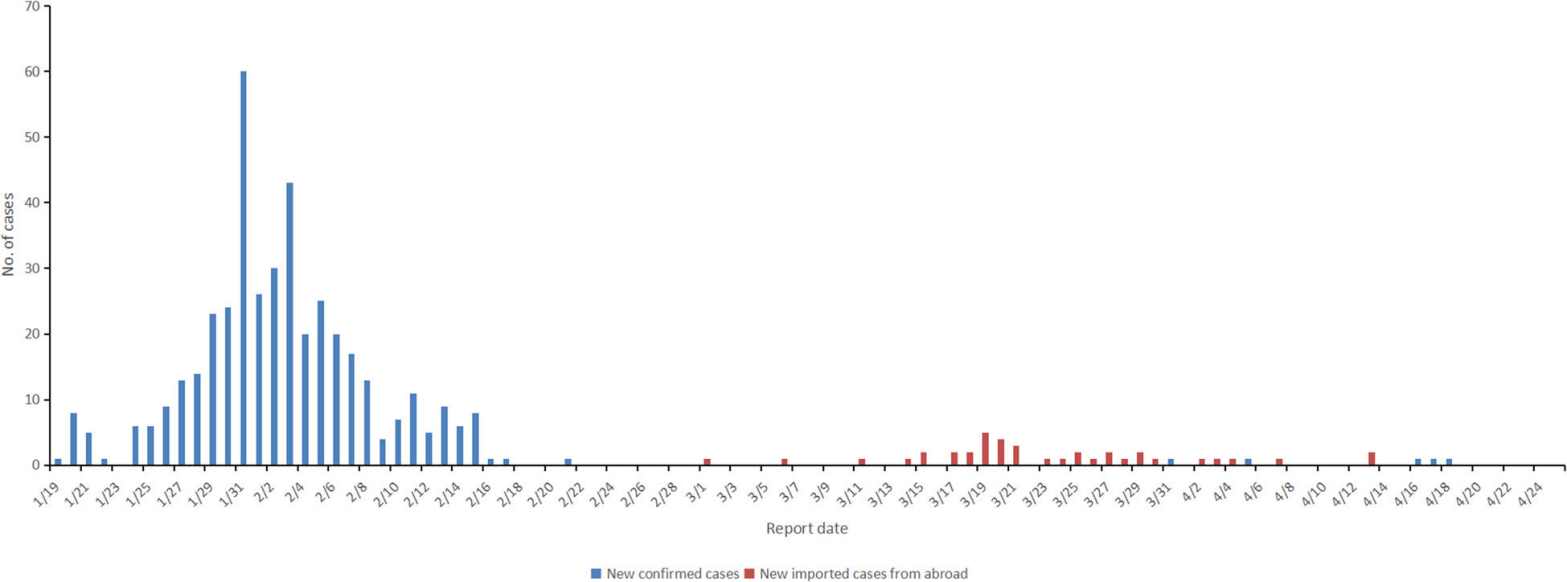
New confirmed cases in Shenzhen

As of April 18, the total number of confirmed cases in Shenzhen was 461, including 309 imported cases from Hubei province, 38 imported cases from other provinces in China, 39 imported cases from abroad, and 75 local cases from multiple modes of infection in Shenzhen (local close contacts, the locals in contact with people in affected areas, and other exposure methods). Figure 9 shows the new confirmed cases in Shenzhen from January 19 to April 25, 2020. Blue indicates the domestic confirmed cases, including imported cases from Hubei and other provinces in China, and the local confirmed cases in Shenzhen. The first confirmed case in Shenzhen was reported on January 19, and the patient came from Hubei. The new confirmed cases in Shenzhen mainly concentrated on the period between January 20 and February 15. Red indicates cases imported from overseas. The first imported confirmed case from the UK was reported on March 1. The imported cases from abroad mainly concentrated on the period between March 14 and April 7. There were no tails on the blue and red epidemiological curves because the prevention and control was effective.

**Fig. 10:**
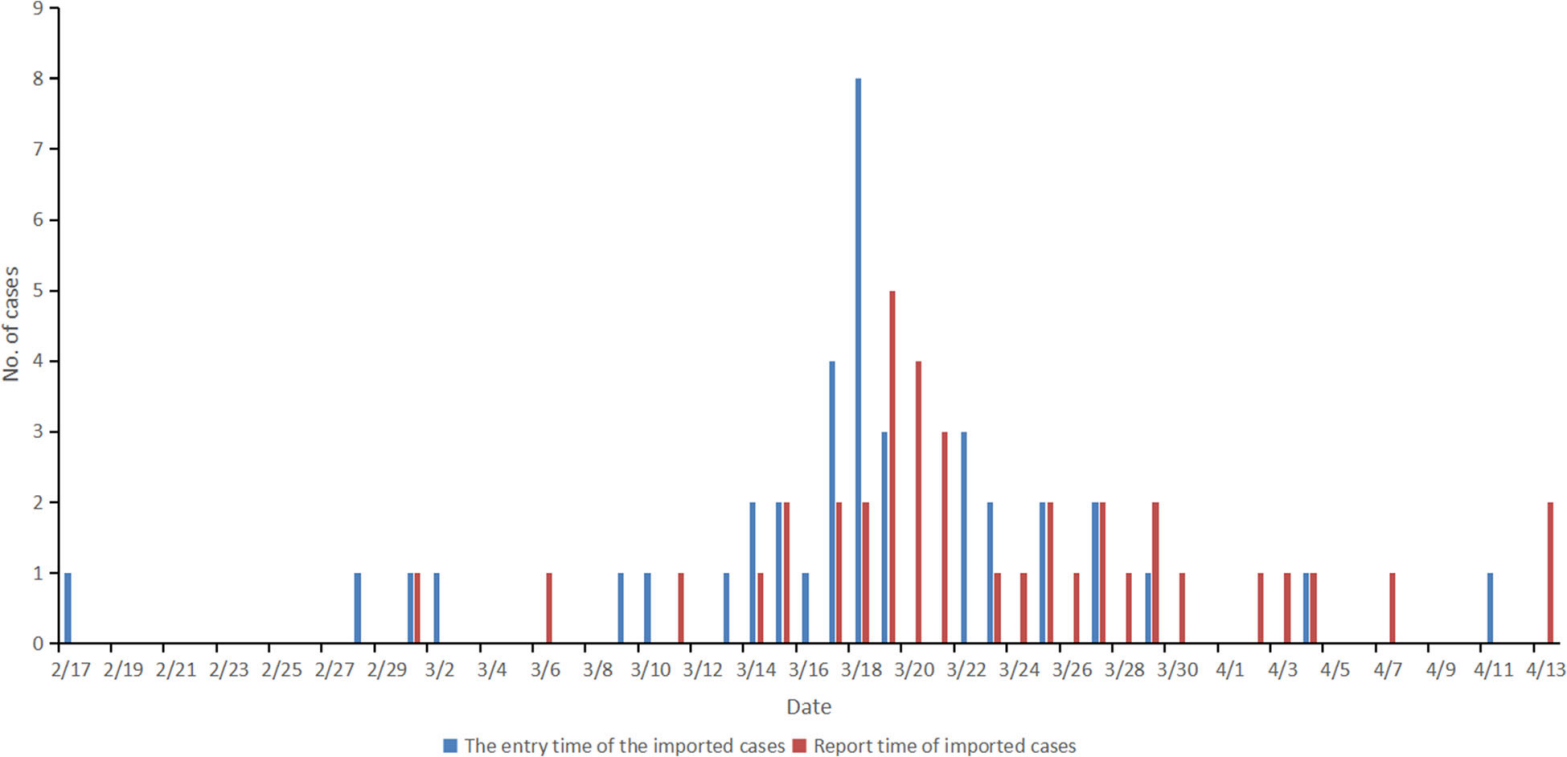
The entry and report time of imported cases in Shenzhen

Figure 10 indicates the entry and report times of imported cases from overseas. Blue indicates the entry time of imported cases, and red indicates the report time of imported cases. The entry and report time ranged from February 17 to April 13, and mainly concentrated on the period between March 10 and March 30. We found that the difference between entry time and report time was small, and the distributions of entry time and report time were similar. Therefore, it is important to use closed-loop management at the port to prevent imported cases and to control domestic relapse.

## Discussion

The findings from our study indicate that 86,844 people from abroad entered Shenzhen, and there were 39 imported cases and 293 closed contacts among them as of April 18, 2020, so the infection rate among people entering was 4.49‰ (95% CI: 3.26‰–6.05‰). The risk of importing infection from the US and UK was higher than for other countries and for regions in Shenzhen. People entering from the US since March 9 or from the UK since March 13 were the high-risk population. All 39 imported cases and 293 close contacts underwent closed-loop management with centralized medical observation when they entered Shenzhen; they received medical treatment in the designated hospital when they were positive on NAT. As of July 17, there have been no new confirmed cases in Shenzhen for 153 days, and the number of confirmed cases, close contacts, and asymptomatic cases was 0. This means the closed-loop management is effective in preventing imported infection and controlling domestic relapse. The difference between the entry time and report time of cases imported from overseas was small, and the distributions of entry time and report time were similar. Therefore, it is important to use closed-loop management at the port to prevent imported cases and control domestic relapse.

Shenzhen is a mega city, immigrant city, and port city with a population of more than 20 million and thus had a high proportion of imported cases from Hubei and other provinces in China [20, 21]. It is also one of the cities with the largest population flow and the highest population density in China, so it is challenging to prevent and control imported COVID-19 infection in Shenzhen. Therefore, we implemented closed-loop management, and it proved to be effective in practice. The closed-loop management also requires additional assistance including the “ACT” community control and prevention mode. A means coordination by administration, C means professional service by a community health service center, and T means cooperation by the trinity community control and prevention mode, including medical staff from community health service centers, community workers, and community police. The “ACT” community control and prevention mode in Shenzhen is effective and highly recognized by WHO experts.

This work has some limitations. Asymptomatic travelers will be missed by symptom-based surveillance, and even if they are tested, some asymptomatic contacts might be missed because of the imperfect sensitivity of the RT-PCR test [22]. The epidemic logical investigations are dependent on individuals’ recall of places visited, people seen, and symptom onset, so the investigation might not have identified all individuals with potential exposure to SARS-CoV-2.

The recent COVID-19 outbreak has been deemed a global health emergency, and internationally the number of confirmed reports has continued to rise [23]. By April 18, 2020, there were more than 2.1 million confirmed cases of COVID-19, and more than 140,000 deaths reported worldwide [12]. Strict containment measures have been effectively implemented throughout China, particularly in infected regions, preventing uncontrolled spread, and the reproduction number has been on a declining trend [24–26]. Every country is closely connected given the level of globalization. In the past several years, multisectoral coordination and collaboration for strengthening of health security has improved substantially [10]. The COVID-19 outbreak requires a robust mechanism of collaboration and cooperation at the global, national, and subnational levels to prevent, detect, and respond effectively [10].

## Conclusions

The closed-loop management has been effective in preventing imported infection and controlling domestic relapse. The distribution of entry time and report time for imported cases overseas was similar. This shows that it is important to implement closed-loop management at the port of entry. Every country is closely connected because of globalization. In order to control the COVID-19 outbreak, we need collaboration and cooperation at the global, national, and subnational levels to prevent, detect, and respond effectively. The findings of our study can provide important reference information and experience for surveillance of imported infection and public health strategy.

## Data Availability

The datasets used and analyzed during the study are available from Shenzhen Municipal Health Commission (http://wjw.sz.gov.cn/yqxx/), Shenzhen government (http://www.sz.gov.cn/), World Health Organization (https://www.who.int/emergencies/diseases/novel-coronavirus-2019/situation-reports/), The U. S Department of State (https://www.state.gov/), The Government of UK (https://www.gov.uk/), The Government of Thailand (http://www.ourweb.info/01/directory/thailand/005/).

## Abbreviations

CDC: Center for Disease Control and Prevention
CI: Confidence intervals
COVID-19: Coronavirus Disease 2019
GBA: Guangdong–Hong Kong–Macao Greater Bay Area
MERS-CoV: Middle East Respiratory Syndrome coronavirus
NAT: Nucleic Acid Testing
RT-PCR: Reverse Transcription–Polymerase Chain Reaction
SARS: Severe Acute Respiratory Syndrome
SARS-CoV-2: Severe Acute Respiratory Syndrome Coronavirus 2
THA: Thailand
UK: United Kingdom
USA or US: United States of America
WHO: World Health Organization
