# Correction to: Study on the COVID-19 infection status, prevention and control strategies among people entering Shenzhen

**DOI:** 10.1186/s12889-021-10895-6

**Published:** 2021-05-12

**Authors:** Xuan Zou, Zi-Qian Xu, Hai-Rui Wang, Bi-Xin Wang, Jian-Fan He, Jing-Zhong Wang

**Affiliations:** 1 Shenzhen Center for Disease Control and Prevention, Shenzhen, China; 2 Shenzhen Bay Laboratory, Shenzhen, China

**Correction to: BMC Public Health 21, 551 (2021)**

**https://doi.org/10.1186/s12889-021-10548-8**

It was highlighted that the original article [1] contained a typesetting error in the authorship, and author Hai-Rui Wang was omitted. The original article has been updated.
